# Adding Prior Information in FWI through Relative Entropy

**DOI:** 10.3390/e23050599

**Published:** 2021-05-13

**Authors:** Danilo Santos Cruz, João M. de Araújo, Carlos A. N. da Costa, Carlos C. N. da Silva

**Affiliations:** 1 Programa de Pós-Graduação em Ciência e Engenharia do Petróleo, Universidade Federal do Rio Grande do Norte, Natal 59064-741, Brazil; 2 Departamento de Física Teórica e Experimental, Universidade Federal do Rio Grande do Norte, Natal 59064-741, Brazil; alexandrecosta17@gmail.com; 3 Departamento de Geofísica, Universidade Federal do Rio Grande do Norte, Natal 59064-741, Brazil; carloscesar@geofisica.ufrn.br

**Keywords:** prior information, entropy, fwi, regularization, inverse problems

## Abstract

Full waveform inversion is an advantageous technique for obtaining high-resolution subsurface information. In the petroleum industry, mainly in reservoir characterisation, it is common to use information from wells as previous information to decrease the ambiguity of the obtained results. For this, we propose adding a relative entropy term to the formalism of the full waveform inversion. In this context, entropy will be just a nomenclature for regularisation and will have the role of helping the converge to the global minimum. The application of entropy in inverse problems usually involves formulating the problem, so that it is possible to use statistical concepts. To avoid this step, we propose a deterministic application to the full waveform inversion. We will discuss some aspects of relative entropy and show three different ways of using them to add prior information through entropy in the inverse problem. We use a dynamic weighting scheme to add prior information through entropy. The idea is that the prior information can help to find the path of the global minimum at the beginning of the inversion process. In all cases, the prior information can be incorporated very quickly into the full waveform inversion and lead the inversion to the desired solution. When we include the logarithmic weighting that constitutes entropy to the inverse problem, we will suppress the low-intensity ripples and sharpen the point events. Thus, the addition of entropy relative to full waveform inversion can provide a result with better resolution. In regions where salt is present in the BP 2004 model, we obtained a significant improvement by adding prior information through the relative entropy for synthetic data. We will show that the prior information added through entropy in full-waveform inversion formalism will prove to be a way to avoid local minimums.

## 1. Introduction

The subsurface image, more specifically, a detailed image of the oil reservoir, is essential in oil and gas exploration and production and requires appropriate data acquisition, processing to remove unwanted information, building a velocity model to use in an appropriate migration algorithm. The quality of the image obtained is, generally, controlled by the subsurface velocity model. When the geology of the area of interest is composed of salt bodies with complex geometrical shapes, the construction of a precise velocity model is more complicated. Full waveform inversion (FWI) is a tool that can provide us with a velocity model with greater precision and resolution. Wang and Rao [1] is, for the first time, applying FWI for the industrial standard reflection seismic data. Through the use of amplitude and travel time content of the acquired seismic data, this technique, theoretically, has the potential to be the most accurate method for the construction of subsurface velocity models [2,3]. Wang and Houseman [4] proposed the joint inversion that uses both the amplitude and travel time data simultaneously, so as to mitigate the ambiguity of reflector geometry and the interval velocities between reflectors.

FWI is a nonlinear and ill-posed data fitting method that usually uses local optimisation methods and, thereforem its solution depends heavily on the initial model. In order to avoid the cycle skipping, the initial model should predict errors in the arrival times less than half the wavelength [3]. One can minimise the issue of non-linearity and the cycle skipping problem with the use of a multi-scale strategy, where we begin from the lowest to the highest frequencies, helping the convergence to the global minimum [5].

The effects of non-uniqueness of the ill-posed inverse problem are usually decreased by the use of regularisation techniques. These regularisation techniques help to stabilise the inversion scheme by incorporating a specific structure or characteristic of the model (e.g., smoothness, sparsity). The most used regularisation scheme is the one that was proposed by Tikhonov and Arsenin [6]. This method incorporated in the inversion scheme aims to find a smooth model that can justify the data. In FWI, in some cases, an l1 model penalty is used as a regularisation strategy to preserve edges and contrasts [7]. However, in some cases, prior information, such as sonic records, stratigraphic data, or geological restrictions, about the model is available. To mitigate the problems of non-uniqueness and stability of the solution, we can use a regularisation scheme that is composed of the model norm and first-order Tikhonov regularisation to act as a smoothing operator, as proposed in [8]. They also suggest that the weighting of the term that is responsible for adding the prior information be done dynamically. This strategy proved to be useful in avoiding local minimums in the FWI. Peters and Hermmann [9] showed a way of including constraints on spatial variations and values ranges of the inverted velocities in FWI.

One way to add prior information to the inversion scheme is through relative entropy. In Thermodynamics, we can introduce the notion of entropy to characterise the degree of disorder of a system. The notion of entropy has been the subject of many controversies and different formulations [10]. Here, we will use entropy just as nomenclature used to restrict the solutions of the inverse problem. In a minimisation problem, when we compare entropy with the model norm, the logarithmic operation will suppress the low-intensity ripples. Thus, the entropy method can deliver images with better resolution in some cases [11]. In other words, adding entropy to the FWI formalism, we introduce a smoothness characteristic in the objective function, which will lead to smoothed solutions that are consistent with the available data [12].

The principle of minimum relative entropy (PMRE) was introduced in [13], and it was first applied in the context of geophysicist by Shore [14] in spectral analysis. Other works applied the PMRE in the seismic deconvolution to make limited band extrapolation [15], diffraction seismic tomography [16], and different geophysical problems, such as inversion of interval velocities, removal of the alias effect, and seismic deconvolution [17]. In the context of the FWI, the entropy concept was applied by Chen and Peter [18], who proposed a misfit function based on entropy regularised optimal transport. da Silva et al. [19] proposed a misfit function for FWI based on Shannon entropy for deeper velocity model updates. None of them made use of prior information and, all of them, in some way, use statistical formalism.

Recently, many works have been developed formulating the FWI in terms of Bayesian formalism. In this sense, Zhu et al. [20] show a Bayesian approach to estimate uncertainty for full-waveform inversion using a priori information from depth migration. Singh et al. [21] propose a robust way to constrain the inversion workflow using per-facies rock-physics relationships derived from well logs. Carvalho et al. [22] show Full-waveform inversion with subsurface fractal information and variable model uncertainties. Zhang and Curtis [23] present the first application of variational full-waveform inversion (VFWI) to seismic reflection data where they imposed realistically weak prior information on seismic velocity.

Usually, when working with entropy, we use probability distribution information or Bayesian formalism, which requires some additional step in the formulation of the problem, such as representing initial and prior models as posterior and prior probability distributions [17]. Our proposal is to add prior information to the FWI using relative entropy without explicitly using the concept of a probability distribution or Bayesian formalism. We will do this in a deterministic and direct way. We present three distinct ways to add prior information to the FWI formalism through relative entropy. We will discuss some aspects of the relative entropy for obtaining velocity models and show how these ways of adding prior information will contribute to recovering the velocity model in areas that are affected by the presence of bodies of salt through a synthetic application on the BP model.

## 2. Theory

Full waveform inversion is an example of a nonlinear ill-posed problem. In general, the solution to this problem is to minimise the discrepancies between the observed and modelled data. From the mathematical point of view, this is a nonlinear problem that is usually being treated as a linearised least-squares problem. However, even the linearised problem still ill-posed and, consequently, several solutions can provide a satisfactory fit between the observed and modelled data.

One way to circumvent this ambiguity of solutions is by introducing prior information adding to the formalism of the inverse problem a terms of regularisation. Thus, we will briefly present the mathematical methodology of the inversion algorithm with the contribution of prior information.

Let *F* be a generic cost function that is given by:

(1) $$F\left(m\right)=\Phi_{d}\left(m\right)+\alpha \Psi_{m}\left(m\right).$$

For FWI, the term $\Phi_{d}\left(m\right)$ is usually constructed through the $L_{2}$ norm of the residue between the modeled and observed data, which is:

(2) $$\begin{array}{c} \Phi_{d}\left(m\right)=\sum_{ns}\frac{1}{2}\left[{\left(d_{obs}-d_{cal}\left(m\right)\right)}^{t}\left(d_{obs}-d_{cal}\left(m\right)\right)\right], \end{array}$$

where $d_{obs}$ and $d_{cal}\left(m\right)$ represent the observed and calculated data vectors, respectively. In our work, we use a time domain approach and each component of these vectors are samples of time domain seismograms recorded at each of the receivers for a seismic source. The misfit function is the result of the sum realized over all ns sources of the seismic acquisition. There is a non-linear dependence on the modeled data and the model parameters, as represented by m. The model parameters are determined by an inverse process that aims to reduce the residue between the modeled and observed data.

In our case, the second term of the cost function will be responsible for adding the prior information (this a prior information can, for example, come from well profiles) to the inversion process. Here, the prior information will be denoted by $m^{r}$. This prior information can be added in different ways to the inversion. In FWI, the model norm with this intention is used in [8]. We use this form for comparison purposes, so we can write Ψ as:

(3) $$\Psi_{m}\left(m\right)=\sum_{i=1}^{N}{\left(m_{i}-m_{i}^{r}\right)}^{2}.$$

The first form that we studied in this experiment was the relative entropy described by [24,25]:

(4) $$\Psi =\sum_{i=1}^{N}m_{i}ln\left(\frac{m_{i}}{m_{i}^{r}}\right),$$

the Equation (4) is the *Kullback–Leibler*’s distance from $m^{r}$ to m. Usually, this equation is used in association with probability distribution. This makes the relative entropy always positive. However, this is only true because the probabilities of the events fall between zero and one [26]. In order to avoid a reformulation of the problem, our idea is to use it in a deterministic way, which is, without using concepts of probability.

Formally, the Kullback–Leibler distance is a pseudo-distance, as it does not satisfy two properties of the metric definition; triangular inequality and symmetry [27]. The fact of non-symmetry led us to our second case study, which will be represented in the equation below:

(5) $$\Psi =\sum_{i=1}^{N}m_{i}^{r}ln\left(\frac{m_{i}^{r}}{m_{i}}\right).$$

**Figure 1:**
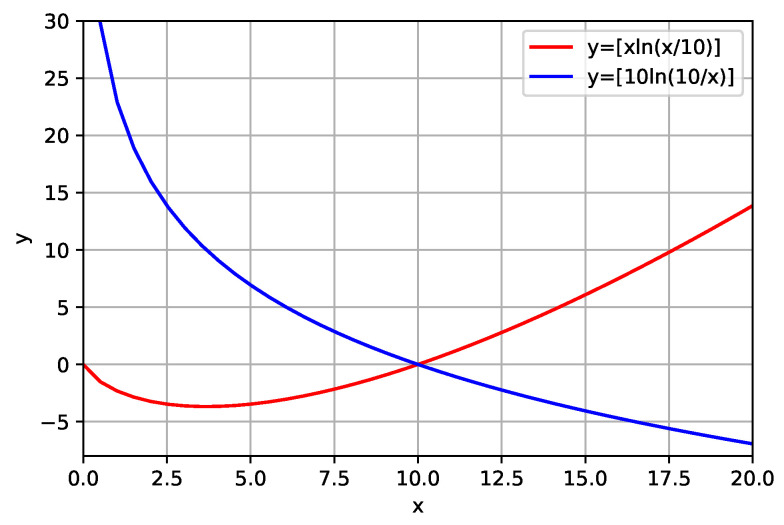
Graphic of the functions y=xln(x/10) (red curve) and y=10ln(10/x) (blue curve). This function is not positive definite. The function y=10ln(10/x) does not present a minimum

It becomes necessary to analyze the behavior of these functions. For this, we simulate a situation in which the prior information was constant ($m^{r}=10$, for example) and plot the graph of the function y=xln(x/10) and y=10ln(10/x), as can be seen in the Figure 1. Analyzing Figure 1, we can infer that Ψ (in Equations (4) and (5)) can present positive and negative values. The graph of Equation (4) (red curve) shows that Ψ will always be negative when the values of the model parameters are less than the parameters of the reference model. The graph of Equation (5) (blue curve) shows that Ψ will always be negative when the values of the model parameters are greater than the parameters of the reference model. In addition, the graph of Equation (5) (red curve) shows that this function does not present a minimum.

This behavior (sometimes positive and negative) in both equations brings an inconvenience to inversion. At one point, we would be minimizing the function at another, maximizing the function.

The simplest way to transform Equation (4) into positive definite is to work with the quadratic form. Thus, we rewrite Equation (4), as follows:

(6) $$\Psi =\sum_{i=1}^{N}{\left[m_{i}ln\left(\frac{m_{i}}{m_{i}^{r}}\right)\right]}^{2}.$$

Analogously, we can change Equation (5) into positive definite taking its quadratic form. Thus, we rewrite Equation (5), as follows:

(7) $$\Psi =\sum_{i=1}^{N}{\left[m_{i}^{r}ln\left(\frac{m_{i}^{r}}{m_{i}}\right)\right]}^{2}.$$

**Figure 2:**
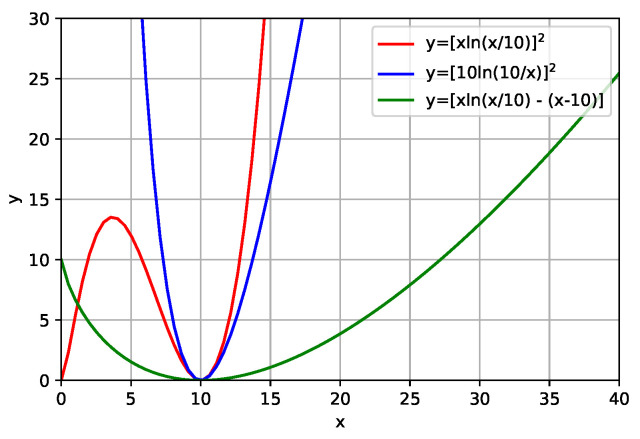
Graphic of the functions $y={\left[xln\left(x/10\right)\right]}^{2}$ (red curve), $y={\left[10ln\left(10/x\right)\right]}^{2}$ (blue curve) and y=xln(x/10)−(x−10) (green curve). The function $y={\left[xln\left(x/10\right)\right]}^{2}$ is positive definite, but it has more than a minimum. The functions $y={\left[10ln\left(10/x\right)\right]}^{2}$ and y=xln(x/10)−(x−10) are positive definite and present only a minimum.

As was done for Equations (4) and (5), we plotted a graph of the function described by Equations (6) and (7), as can be seen in Figure 2. Analyzing Figure 2, it can be seen that Equation (6) is positively defined. However, in the example that is illustrated in Figure 2, we can observe the presence of two minimums. This leads us to interpret that the use of Equation (6) in FWI can increase the problem of local minimums (this will be exemplified in numerical applications). Also analyzing Figure 2, we can expect that Equation (7) presents the characteristics that are favorable to the inversion process, which is, it is a definite positive function and that only presents a minimum.

Finally, we can add the prior information to FWI with the axiomatic form that is given by (8) [28,29]:

(8) $$\Psi =\sum_{i=1}^{N}\left[m_{i}ln\left(\frac{m_{i}}{m_{i}^{r}}\right)-\left(m_{i}-m_{i}^{r}\right)\right],$$

the Equations (4) and (8) are similar. However, Equation (8) has a term referring to the difference of the models that leaves it with the characteristic that we expect (definite positive function), as can be seen in Figure 2 in a green curve.

If we minimize the objective function in the classical way, we obtain a system of equations that can be expressed as:

(9) $$H_{F}\Delta m=-G_{F},$$

where $H_{F}$ and $G_{F}$ represent the Hessian and gradient of cost function, respectively. In this case, the gradient represents the sum of two terms. If the Ψ function is the model norm (Equation (3)), we have:

(10) $$G_{F}=J^{T}\left(d_{obs}-d\left(m\right)\right)+2\alpha \left(m_{i}-m_{i}^{r}\right).$$

For the Ψ function to be represented by Equation (6), the gradient will be represented, as follows:

(11) $$G_{F}=J^{T}\left(d_{obs}-d\left(m\right)\right)+2\alpha \left[\left(m_{i}ln\left(\frac{m_{i}}{m_{i}^{r}}\right)\right)\left(ln(\frac{m_{i}}{m_{i}^{r}})+1\right)\right].$$

When the Ψ function that is represented by Equation (7) is used, the gradient expression will be given by:

(12) $$G_{F}=J^{T}\left(d_{obs}-d\left(m\right)\right)-2\alpha \left[\left(m_{i}^{r}ln\left(\frac{m_{i}^{r}}{m_{i}}\right)\right)\left(\frac{m_{i}^{r}}{m_{i}}\right)\right].$$

In case the Ψ function used is the one represented in Equation (8), the gradient will be described, as follows:

(13) $$G_{F}=J^{T}\left(d_{obs}-d\left(m\right)\right)+\alpha \left[m_{i}ln\left(\frac{m_{i}}{m_{i}^{r}}\right)\right].$$

The term J that is present in the gradient expressions is the sensitivity matrix. The sensitivity matrix is composed of the derivatives of the modeled data with respect to the model parameters (J=∂d(m)/∂m). The elements of J are not explicitly calculated because they demand a high computational cost. For this reason, the adjoint formulation [30] is used for this purpose.

Asnaashari et al. [8] showed that we should work with a dynamic weighting of the term regularisation. The basic idea of this methodology is to help the inversion process to converge to the global minimum of the objective function by increasing the importance of prior information at the beginning of the process and gradually decreasing the penalty term weighting until, in the final iterations, the convergence driven by the term of the data. In this work, the α=μγ parameter is a dynamic weighting of the term regularisation and it has the role of decreasing the weight of the entropy term over the iterations. We built the dynamic term (γ) from the ratio of the gradients, as can be seen in Equation (14):

(14) $$\gamma =\frac{\sum_{i=1}^{M}{\left(\nabla \Phi_{i}\right)}^{2}}{\sum_{i=1}^{M}{\left(\nabla \Psi_{i}\right)}^{2}},$$

where $\Phi_{i}$ and $\Psi_{i}$ are the elements of the gradients vectors of misfit and regularise, respectively. In our tests, as will be seen in numerical applications, the γ value proved to be inadequate (the initial value was large) and it needed to be adjusted. We made this adjustment using the μ parameter.

We calculated the gradients of the terms of those that are responsible for adding the prior information easily and directly added to the data gradient. The term of the Hessian matrices, which is composed of the second derivative of misfit function and relative entropy, is not explicitly resolved in this paper. We calculated the hessian using a limited quasi-newton method that is known in the literature as L-BFGS-B. The routine that was proposed by [31] considers that the inverse Hessian matrix is non-diagonal and roughly obtains its elements from the gradient vectors and previous models by performing a line search that satisfies Wolfe’s conditions.

## 3. Numerical Tests

We only worked with the acoustic case (i.e., a P velocity model) and considered the homogeneous density distribution. We also considered a regular grid with 12.5 m spacing, which is used in both modelling and inversion. The data that were observed and modelled in time were obtained from the acoustic wave equation through finite-difference modelling, where an eighth order approximation for the Laplacian operator and a second-order approximation for the time derivative were considered. A CPML absorption boundary layer was employed to avoid boundary reflection [32,33]. The absorbing layer was applied to all sides of the model, using a width of 40 cells. The FWI worked here was performed in the time domain using all spectrum frequencies.

In this section, we will show the contribution of prior information added to the FWI through relative entropy. Therefore, we chose using the first and second part of the BP 2004 benchmark [34]. For the first part, the acquisition geometry consisted of 475 hydrophones distributed along a straight line 12.5 m deep, with 12.5 m spacing between each receiver. For the shots, 15 sources spaced 395 m arranged in line with 25 m deep. For all shots, a Ricker wavelet source with a central frequency of 10 Hz was used and the time record was 5.0 s. For the second part, the geometry acquisition is similar to that used in the first part, but the central frequency of Ricker was 12 Hz with a record time of 6.5 s.

**Figure 3:**
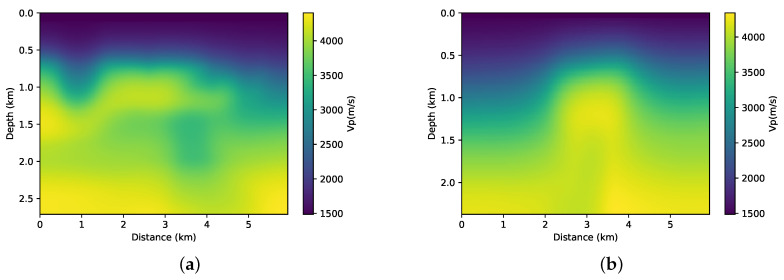
Illustration of the initial models used as an initial model to FWI. (**a**) Smoothing of the first part from the BP model. (**b**) Smoothing of the second part from the BP model.

Given that FWI is usually treated as an iterative process, an initial velocity model is required. For example, this model may be the result of a tomography that is based on the times of first arrivals and reflected events. For this work, we perform the smoothing of the real model (Figure 3), and we use it as an initial model in the FWI process.

**Figure 4:**
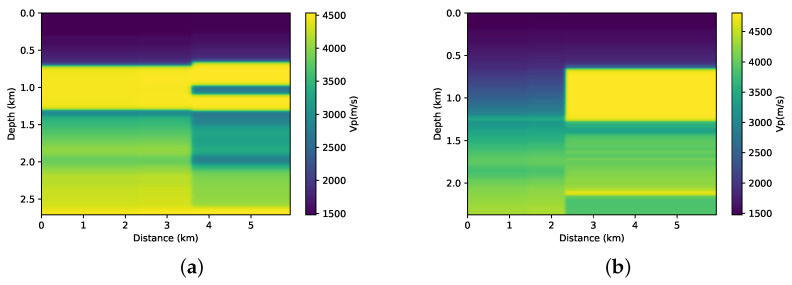
The prior models built by linear interpolation between the values and extrapolation outside from wells that will be added in the FWI. (**a**) Prior model of the first part from BP model, (**b**) Prior model of the second part from BP model.

For this study, we assume that we have information from exploration wells. The velocities profile are measurements that provide a good measure of the local depth velocity. Thus, we will use these sonic profiles to build our a priori information model. A linear interpolation was made between the two wells. In the other regions, we use an extrapolation of the well’s velocity profile. We also apply a slight smoothing to this a priori model of velocity. We can see this interpolated model in Figure 4. Although not geologically significant, this model contains some travel time information, and it will be considered an a priori velocity model and incorporated into the FWI through the relative entropy of the model.

**Figure 5:**
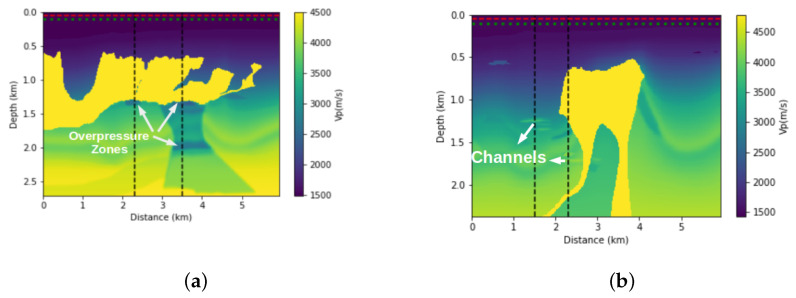
True $V_{p}$ velocity model, which are parts of the BP model and the scheme of acquisition. The red-dashed line represents the position of the receivers, while the green line represents the position of the sources. (**a**) First part from the BP model, the white arrows illustrate the target zones (overpressure zones) and the black-dashed lines represent the position of the two wells that cross overpressure zones. (**b**) Second part from the BP model, the white arrows illustrate the target zones (channels) and the black-dashed lines represent the position of the two wells that cross the channels.

**Figure 6:**
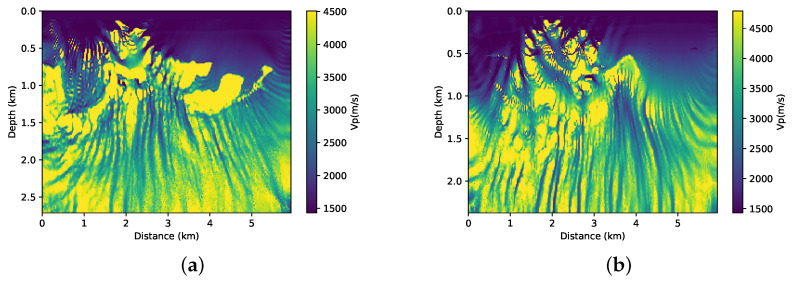
(**a**) FWI results using the smoothed model and without prior information (α=0) (**a**) in first part of BP model and (**b**) in second part of BP model.

First, we performed the inversion without adding prior information on both parts of the BP model that are illustrated in Figure 5a,b. This means that, in Equation (1), α=0. We used the initial models that are shown in Figure 3a,b. The results of the FWI for each of the cases are shown in Figure 6a,b. Clearly, in both cases, the conventional FWI (without any type of regularisation) converges to a local minimum. Asnaashari et al. [8] discussed some differences between the prior and initial models in the inversion procedure. In this case, the smoothed model that is shown in Figure 3 and the a priori model shown in Figure 4 have only part of the real model information. For the FWI result to converge to the desired result, both of the models must be used in a complementary way.

Given the obtained results, we will add the prior information to the FWI formalism in four different ways, as shown later on.

### 3.1. Model Norm

**Figure 7:**
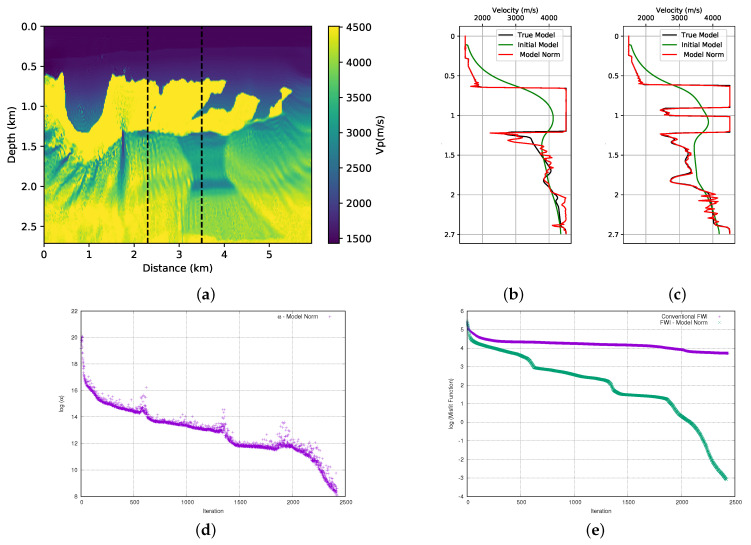
First part of BP model; (**a**) FWI result with adding prior information through the model norm; (**b**) profile in well at position x= 2.3 km, (**c**) profile in well at position x= 3.5 km and (**d**) dynamic term (α) progress, (**e**) misfit data function progress (logarithmic natural base scale).

**Figure 8:**
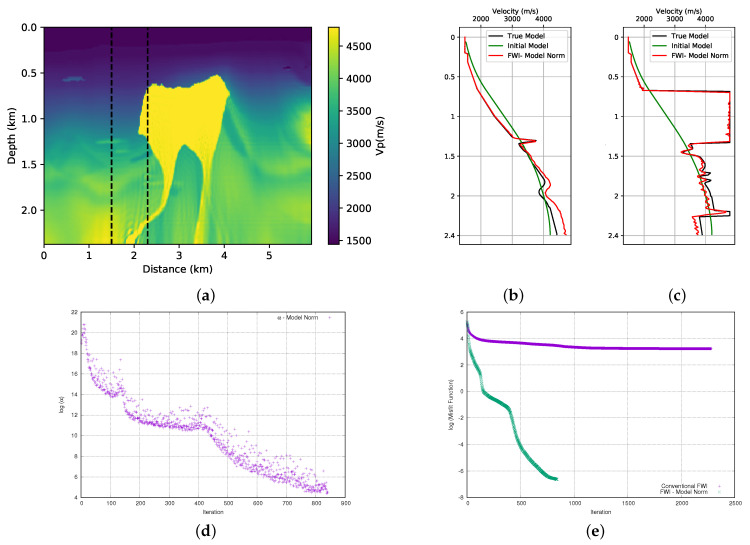
Second part of BP model; (**a**) FWI result with adding prior information through the model norm; (**b**) profile in well at position x= 1.5 km (**c**) profile in well at position x= 2.3 km, (**d**) dynamic term (α) progress (**e**) misfit data function progress (logarithmic natural base scale).

In this section, we add the prior information to the FWI using Equation (3). This method was used by [8] to incorporate the prior information in the FWI. Here, as previously mentioned, we will use the results obtained here to compare with the entropy methods that are the focus of this work. Therefore, we performed the FWI for both models (first and the second parts of the BP model), adding the prior information that is shown in Figure 4. The initial models used are those that are represented in Figure 3. Figure 7a and Figure 8a show the results. When we compare these results with those that are obtained without adding prior information (Figure 6a,b), we observe an improvement in the quality of the FWI result. We observed that, in general, the body of salt was recovered in both models (although the first part of the BP model presents a small problem on the left side of the well positioned at x=2.3 km). For a more detailed quality control, we can see the profiles in the positions of the wells in Figure 7b,c for the first part of the model, and Figure 8b,c for the second part of the model. By analysing the profiles, we can confirm that the addition of prior information in FWI through the model norm provides a good result.

A crucial point for the success of adding prior information in FWI scheme is the choice of the alpha parameter. In this work, as described in the theoretical section, the α parameter is the product of two terms. The first is a dynamic term (γ), as calculated from Equation (14). Using the model norm, the γ parameter initiated the inversion process equal to 6 × $10^{+17}$ and 7.6 × $10^{+17}$ for the first and second parts of the BP model, respectively. The initial value of the γ parameter proved to be inadequate and it needed to be adjusted. Consequently, we use a second term, the μ parameter to adjust the weight of the regularization term. After several tests, we found that the parameter should be 3.5 × $10^{-10}$ and 3 × $10^{-10}$ for the first and second part of the BP model, respectively. Once the values of the μ and γ parameters are found, determining the α parameter is straightforward. The evolution of the α parameter for each model can be seen in Figure 7d and Figure 8d. Note that the α values are on the log (natural base) scale. We can see the expected behavior for the weight (α) given to the model norm term in Figure 7d and Figure 8d. We observed that there was a sharp drop at the beginning of the inversion. As the model is updated, these terms will decrease and the seismic data will conduct the inversion.

The misfit data curve is shown in Figure 7e for the first part of the model and Figure 8e for the second part of the model. For all of the tests that we performed, we used a small stop criterion to ensure that the data adjustment was as large as possible, which resulted in a large number of iterations. For the stopping criterion, a tolerance limit of 9 × $10^{-9}$ was established in the total value of the model update (total gradient). The misfit data curves show a great difference between the convergence of conventional FWI (without any type of regularisation or prior information) and FWI with the addition of prior information through the model norm. Even with several iterations, the conventional FWI cannot reduce the misfit data, while the FWI with prior information shows a very sharp drop at the beginning of the iterations that continues until it reaches a relatively satisfactory result.

### 3.2. First Case: Kullback-Leibler’s Distance

**Figure 9:**
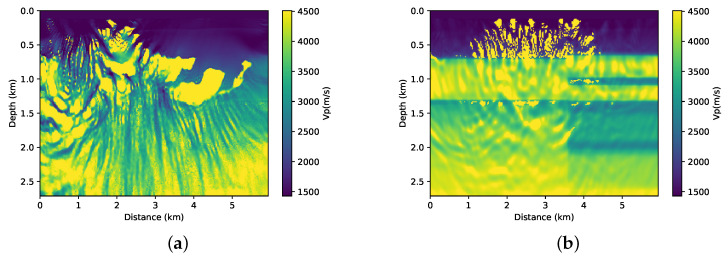
FWI results with adding prior information through the quadratic form of relative entropy for the first part of BP model. (**a**) initial α=2 × $10^{7}$ (**b**) initial α=3 × $10^{7}$.

As mentioned earlier, our proposal is to add priori information to the FWI formalism through relative entropy. The first attempt to add prior information to FWI through entropy would be with the use of the relative entropy that is described by Equation (4). To use it, it would be necessary to represent entropy as a probability distribution function. This implies a normalization constraint, that is, 0≤p≤1 [17]. For the addition of a priori information to be done in a simple and direct way, we will do this in a deterministic way. Thereby, the Equation (4) is not adequate as previously discussed. Equation (4) is not positively defined in any interval. The way to get around the problem that was brought by Equation (4) was to work with its quadratic form represented by Equation (6). Thus, we started the tests in the first part of the BP model using α=2 × $10^{+7}$. Even with the addition of prior information, the result converged to a local minimum and it is far from the expected result, as can be seen in Figure 9a. A natural idea would be that the initial weight given to the entropy term is inadequate. Consequently, we increased the value of this initial weight to α=3 × $10^{+7}$ and with few iterations we obtained the result that is illustrated in Figure 9b. In this result, which is still a local minimum, we note that the FWI is leading the solution for the prior model. Although we performed other tests with intermediate values for alpha, we did not achieve the desired success for this case.

**Figure 10:**
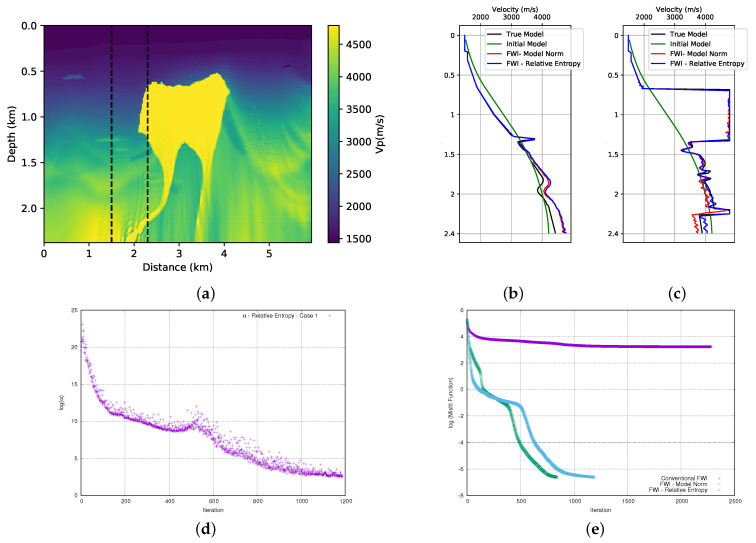
(**a**) FWI results of adding prior information through the quadratic form of the relative entropy in the second part of BP model; (**b**) velocity profile at position x=1.5 km, (**c**) velocity profile at position x=2.3 km and (**d**) α parameter evolution (note this curve is shown in logarithmic natural base scale), and (**e**) misfit data function progress (logarithmic natural base scale).

Even with the failure in the first attempt to add the quadratic form of entropy relative to the formalities of the FWI, we performed the test in the second part of the BP model. Figure 10a shows the result. The analysis of the result that is illustrated in Figure 10a shows that in this case the addition of prior information through the quadratic form of the relative entropy enabled the FWI to converge on a satisfactory solution. When comparing the result that was obtained with the model norm (Figure 8a), a visible similarity is observed. A more detailed analysis of the position of the wells confirms this similarity when we compare the adjustment at position x=1.5 km. The model norm (red curve) and the relative entropy (blue curve) both provide equivalent results, as can be seen in Figure 10b. However, when we see the position x=2.3 km (Figure 10c), the adjustment that is provided by the relative entropy proved to be slightly better to the model norm, mainly in the deep part of the model. The initial alpha for this case was α∼8 × $10^{7}$ (in this case, γ∼2.7 × $10^{17}$ and μ=3 × $10^{-10}$) and its evolution can be seen in Figure 10c. We observe that there is a marked decrease in the weight given to the end of the relative entropy, which allows us to avoid giving too much importance to the entropy term by ensuring that an adequate contribution of the prior information is maintained throughout the iterations.

Figure 10e illustrates the data misfit. As seen in the case of the model norm (green curve), the addition of prior information through relative entropy (light blue curve) causes the FWI to drastically reduce the misfit of the data, leading to a low misfit result. Even though the relative entropy leads to an inversion around iteration 470 through a path of misfit greater than that of the model norm, the values of the final misfit are close.

The failure in the first part and the success in the second part of the BP model led us to conclude that the initial reasoning made through the analysis of the graph that is shown in Figure 2 was correct. In other words, the quadratic form of the relative entropy can somehow increase the problem of local minimums, but, depending on the path that the inversion process takes, we can find the global minimum.

### 3.3. Second Case: Kullback-Leibler’s Distance—Symmetric Form

**Figure 11:**
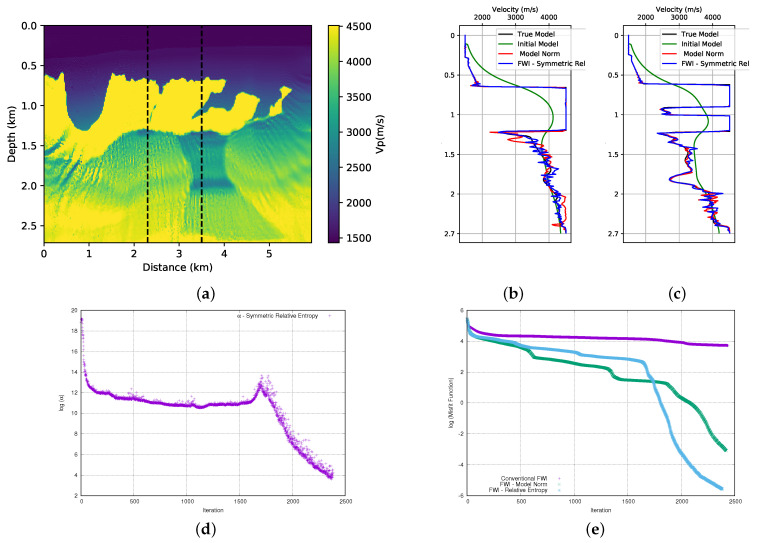
(**a**) FWI result with adding prior information through the quadratic form of the relative entropy in first part of BP model; (**b**) velocity log at position x=2.3 km; (**c**) velocity log at position x=3.5 km; (**d**) α parameter evolution (Note this curve is shown in logarithmic natural base scale); and, (**e**) misfit data function progress (logarithmic natural base scale).

**Figure 12:**
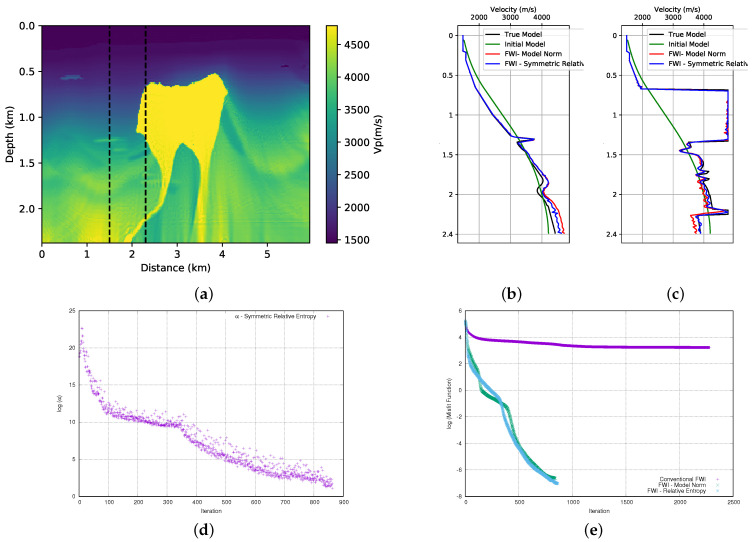
FWI result with adding prior information through the quadratic form of the relative entropy in second part of BP model; (**b**) velocity log at position x=1.5 km; (**c**) velocity log at position x=2.3 km; (**d**) α parameter evolution (Note this curve is shown in logarithmic natural base scale); and, (**e**) misfit data function progress (logarithmic natural base scale).

There are few applications in the literature for the symmetric form of relative entropy that is shown in Equation (5). Probably the reason for this is the fact that this equation does not have a minimum region, as mentioned in the theoretical section. Therefore, our second proposal is to add the priori information in FWI is through the quadratic form that is represented in Equation (7). Analogously to the previous case, we will also add priori information to the problem directly, without using a probability distribution formalism. First, we performed the tests on the first and second parts of the BP model. The result can be seen in Figure 11a and Figure 12a. We can observe, in both cases, that the addition of prior information through the use of the quadratic form of the symmetric relative entropy allows the FWI to provide satisfactory results.

We compare the result of the FWI (for the first part of the model) using the symmetrical form of the relative entropy (Figure 11a) with that obtained with the model norm in Figure 7a. We observe that the addition of prior information through the symmetric relative entropy provides a better result on the left-hand side of the model. We can confirm the quality of the result by looking at the velocity profiles in the well positions, see Figure 11b,c. We can observe that, for the well at position x=2.3 km (Figure 11b), the result of the FWI with the addition of prior information through the symmetric form of the relative entropy when compared to the use of the model norm provides a better approximation of the real value in the region below the salt. For the well at position x=3.5 km (Figure 11c), the results of the FWI with the symmetric relative entropy and with the model norm provide equivalent adjustments. As for the second parts of the model, the result of the FWI with the symmetric relative entropy (Figure 12a) is visibly equivalent to the result that was obtained with the model norm (Figure 8a). However, when we observe the velocity profiles in the well positions that are shown in Figure 12b,c, we can see that adding the symmetrical case of the relative entropy in the FWI provides a slightly better fit in the deeper part of the model than the model norm.

After several tests, we concluded that the initial value of the α parameter for the first part of the BP model should be equal to 1.4 × $e^{6}$ (in this case, γ∼2.8 × $10^{17}$ and μ=5 × $10^{-10}$). Its progression can be seen in Figure 11d. For the second part of the BP model, the initial value of α parameter was equal to 1.3 × $e^{6}$ (in this case, γ∼2.6 × $10^{17}$ and μ=5 × $10^{-10}$, and we show its evolution in Figure 12d. As previously mentioned, we observed that the α parameter provides an adequate balance between the term of the data and the prior information added through the relative entropy. The misfit data curve for the first part of the model is illustrated in Figure 11e. The relative entropy (blue curve) that is added to the FWI provides a result with a better fit than with the model norm (green curve) and conventional FWI (purple curve). For the second part of the model, we can see the misfit data curve in Figure 12e. We observe that the addition of information through the relative entropy (light-blue curve) to the FWI also provides an extensive decay in the misfit data when compared to the classic FWI (purple curve). When comparing the misfit data of FWI with the model norm (green curve), we see that the final adjustment is close, although the misfit with relative entropy is a little better.

### 3.4. Third Case: Axiomatic Form

**Figure 13:**
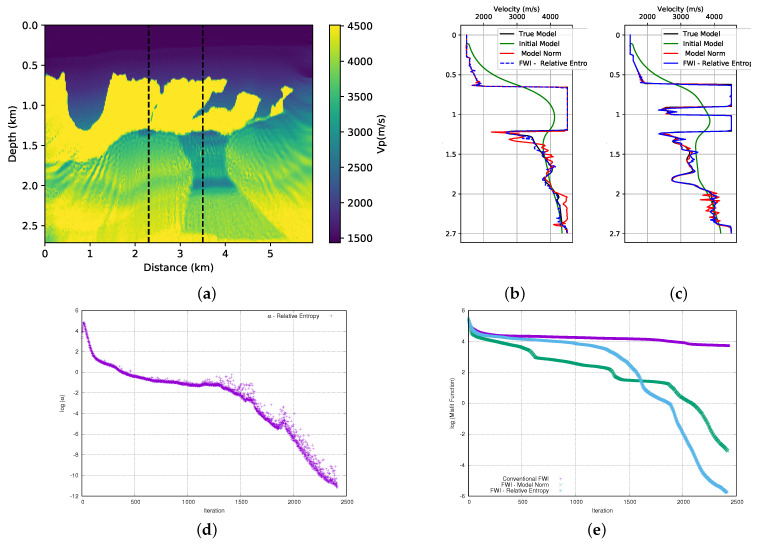
(**a**) FWI result of adding prior information through the relative entropy in the first part of the BP model: (**b**) velocity log at position x=2.3 km; (**c**) velocity log at position x=3.5 km; (**d**) α parameter evolution (Note this curve is shown in logarithmic natural base scale); and, (**e**) misfit data function progress (logarithmic natural base scale).

**Figure 14:**
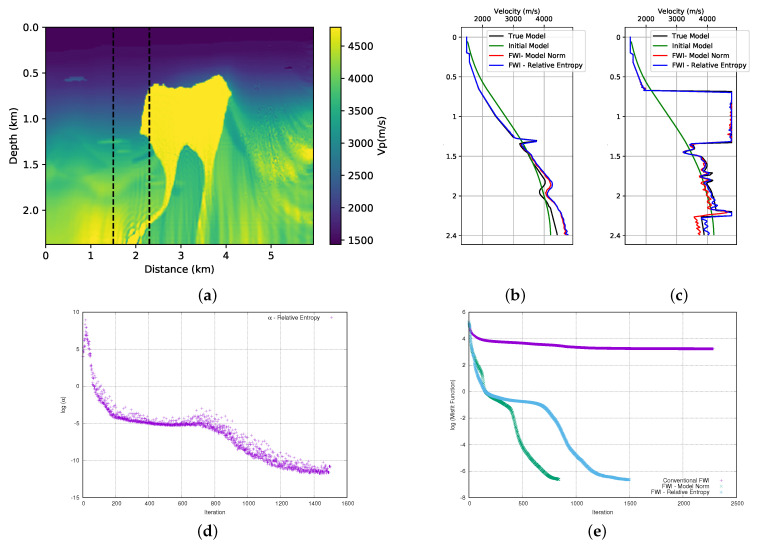
(**a**) FWI result with adding prior information through the relative entropy in second part of BP model: (**b**) velocity log at position x=1.5 km; (**c**) velocity log at position x=2.3 km; (**d**) α parameter evolution (Note this curve is shown in logarithmic natural base scale); and, (**e**) misfit data function progress (logarithmic natural base scale).

Finally, our third proposal to add relative entropy in FWI is the Axiomatic form. We also use this form to add priori information to the FWI. Its form is described in the Equation (8). The advantage of the relative entropy described by Equation (8) is that it is positively defined, which makes the application straightforward without the need for any adjustments. Thus, we used Equation (8) and performed the FWI in the first of the BP model, and we show the result in Figure 13a. As in the previous cases, when we compare the result of the FWI with the use of the model norm (Figure 7a) to add the prior information, the relative entropy (8) added to the FWI provides a slightly better quality result on the left-hand side of the model. The analysis of the velocity profiles in the well positions show that the FWI with the addition of the relative entropy (blue curve) as compared to the FWI with the addition of the model norm (red curve) provides an adjustment that is closer to the desired in the well in the position x=2.3 km (Figure 13b) and a similar adjustment at position x=3.5 km (Figure 13c). We also performed FWI in the second part of BP model, and it can be seen in Figure 14a. In this case, we can see the similarity of the results when we compare this result with that obtained using the model norm (Figure 8a). The analysis of the velocity profiles in the well positions shows an equivalent result in the well position at x=1.5 km (Figure 14b) and, in the position x=2.3 km, we observe a slightly better result of the FWI with the relative entropy (Figure 14c).

After several tests, we concluded that, for this case, the weight value of the entropy term should start at α=25 (in this case, γ∼2.8 × $10^{4}$ and μ=9 × $10^{-4}$) for the first part of the BP model. The evolution of the α parameter can be seen in Figure 13d. For the second part of the BP model, we conclude that the alpha parameter should be α=50 (in this case, γ∼5.6 × $10^{4}$ and μ=9 × $10^{-4}$) and its evolution can be seen in Figure 14d. In Figure 13e and Figure 14e, we see the misfit data curve for the first and second parts of the model, respectively. We observe that in the case of the first part of the model, the FWI with the relative entropy (light-blue curve) starts the inversion in a path that is very close to the conventional FWI (purple curve). However, around the iteration 1350, the data adjustment begins to improve significantly, finishing the inversion at a lower misfit data value than the FWI with the model norm (green curve). For the second part of the model, we observe that the FWI with the relative entropy (light-blue curve) starts the inversion in a path that is very close to the FWI with the model norm (green curve). However, at iteration 200, the FWI with relative entropy takes a path of a larger misfit, so that more iterations are needed to obtain a misfit data result that is close to that obtained by the FWI with the model norm.

### 3.5. Discussion

We have shown that the addition of a priori information to the FWI scheme can be an effective strategy for driving the inversion process to converge toward the global minimum. Here, we have incorporated the priori information in different ways and, to quantify the accuracy of the inversion results for each one, we have computed the normalized model misfit using the following equation:

(15) $$\epsilon =\frac{{\left[\sum_{i=1}^{n}{\left(m_{i}^{true}-m_{i}^{inv}\right)}^{2}\right]}^{1/2}}{{\left[\sum_{i=1}^{n}{\left(m_{i}^{true}\right)}^{2}\right]}^{1/2}},$$

where $m^{true}$ is the true model and model and $m^{inv}$ is the inverted model using an FWI method [35,36]. An ϵ close to 0 means low error. The ϵ values are shown in the Table 1.

In the analysis of the results that are presented in Table 1, we observe that axiomatic form (third case) We note that the third case provides the model with the smallest error in both cases. Although it does not provide the smallest error in the model, our proposal to use the quadratic form of the symmetric form of the relative entropy (second case) is also robust. It is possible to see that the model error with this strategy is less than using the model norm. Finally, it is possible to observe that our proposal to use the quadratic form of relative entropy (first case), although its result depends on the inversion path, can also provide a good result. For the first part of the model, we were not successful in our tests, but we calculated the error for the results that are shown in Figure 9a,b, respectively. For the second part of the model, we can observe that the model error is less than the conventional case.

## 4. Conclusions

In this work, we propose adding the relative entropy in the FWI formalism. We use relative entropy to include priori information in the FWI to reduce the difficulty of the uniqueness of the solution in this kind of inverse problem. The addition of the prior information was done in a deterministic way, which is, it was done in a direct way, avoiding the formulation in terms of a probability distribution. We have applied this scheme in two regions of the BP model, which presents a complex lateral velocity variation due to the halokinesis of salt layers. The numerical tests show the quality improvement in the result that was obtained when compared with the conventional FWI. In addition to the visual analysis, we calculated the misfit model to show the improvement that was brought by our proposal.

We present three different ways of introducing prior information through entropy to the FWI formalism: the literature as Kullback–Leibler’s distance and its symmetrical form and an axiomatic form. In the first case, we show that in the deterministic approach, the Kullback–Leibler’s distance is not positively defined in its entire domain. To avoid this misfortune, we suggest using its quadratic form. We have seen that this quadratic form will not always help to solve the local minimum problem. We graphically illustrate that the function described by the quadratic form of the Kullback–Leibler’s distance has two regions of minimum. Therefore, the result will depend on the path taken by the FWI throughout the iterations: while it was not possible to obtain a satisfactory solution for the first part of the model after several tests, for the second part of the model, the result was satisfactory.

The second case was the symmetrical form of Kullback–Leibler’s distance. We graphically illustrate that this function is also not positively defined, which makes it difficult to define the problem as a maximization or minimization. In addition, the symmetrical form has no minimum region. To avoid these inconveniences, we propose the use of its quadratic form. We have shown graphically that this quadratic shape has characteristics that can help the FWI to avoid local minimums. In addition to being positively defined, it presents a minimum region. The addition of previous information in the FWI through this quadratic form enables the FWI to deliver a satisfactory result for both cases.

The third case that we studied was the addition of prior information through an axiom of relative entropy. Graphically, we show that this shape is positively defined and it has a minimum region. These features allow this form to be used to add information prior to the FWI formalism in a straightforward manner. The results of the FWI with this regularization scheme were also satisfactory for both models.

The FWI results that were obtained using the relative entropy were compared to the result with the model norm. We observed that for the first part of the BP model, the FWI result with real entropy is slightly better (mainly on the left-hand side of the model). This fact is corroborated by the analysis of the velocity profiles in the position of the wells. We saw that the FWI result with the relative entropy provides an adjustment closer to the desired result when compared to the FWI result with the model norm. In addition, we saw that the misfit data are less when we add prior information through entropy. For the second part of the BP model, the results are visibly similar. We have seen that the adjustment of the data is close, although the first and third cases of relative entropy require a little more iteration. However, when comparing the well profiles, we observed that the adjustment of the FWI with the addition of prior information through entropy provides a better fit early in the deepest region of the model.

## Figures and Tables

**Table 1:** Misfit model of the FWI results. (∗) This is the model misfit for the result that is illustrated in Figure 9a, while (∗∗) is the model misfit for the result illustrated in Figure 9b.

Strategy	First Part	Second Part
	ϵ	ϵ
Conventional FWI	0.6389	0.4415
FWI + Model Norm	0.0114	0.1132
Our proposal: FWI + First case	0.04963 ${}^{*}$/0.0091 ${}^{**}$	0.1415
Our proposal: FWI + Second case	0.0063	0.0968
Our proposal: FWI + Third case	0.0025	0.0876

## Data Availability

The data presented in this study are available on request from the corresponding author.

## Abbreviations

The following abbreviations are used in this manuscript:

FWI	Full Waveform Inversion
